# Clinical presentation, case management and outcomes for the first 32 COVID-19 patients in Nigeria

**DOI:** 10.11604/pamj.supp.2020.35.2.23262

**Published:** 2020-05-06

**Authors:** Abimbola Bowale, Akin Abayomi, Jide Idris, Sunday Omilabu, Ismail Abdus-Salam, Busayo Adebayo, Folarin Opawoye, Ore Finnih-Awokoya, Emmanuella Zamba, Hussein Abdur-Razzaq, Olufemi Erinoso, Tosin Onasanya, Patrick Ramadan, Sam Nyenyi, Evaristus Aniaku, Muhammad Balogun, Oyeladun Okunromade, Olusola Adejumo, Sunday Adesola, Tope Ogunniyan, Mobolanle Balogun, Akin Osibogun

**Affiliations:** 1 Mainland Hospital, Yaba, Lagos, Nigeria; 2 Lagos State Ministry of Health, Lagos, Nigeria; 3 College of Medicine University of Lagos, Lagos, Nigeria; 4 Lagos State University Teaching Hospital, Lagos, Nigeria; 5 Lagos University Teaching Hospital, Lagos, Nigeria; 6 Office of the Senior Special Assistant to the Governor, Lagos, Nigeria; 7 Lagos State Primary Health Care Board, Lagos, Nigeria; 8 World Health Organization, Nigerian Office, Lagos, Nigeria; 9 Nigerian Centre for Disease Control, Lagos, Nigeria; 10 African Field Epidemiology Network, Lagos, Nigeria

**Keywords:** COVID-19 presentation, clinical outcomes, Nigeria, retrospective study

## Abstract

**Introduction:**

Success in curtailing the pandemic coronavirus disease (COVID-19) depends largely on a sound understanding of the epidemiologic and clinical profile of cases in a population as well as the case management approach. This study documents the presenting characteristics, treatment modalities and outcomes of the first 32 COVID-19 patients in Nigeria.

**Methods:**

This retrospective study used medical records of the first 32 patients admitted and discharged from the Mainland Hospital, Lagos State, southwest Nigeria between February 27 and April 6, 2020. The outcomes of interest were death, promptness of admission process and duration of hospitalization.

**Results:**

The mean age of the patients was 38.1 years (SD: 15.5) and 66% were male. Three-quarters (75%) of the patients presented in moderately severe condition while 16% were asymptomatic. The most common presenting symptoms were fever (59%) and dry cough (44%). The mean time between a positive test result and admission was 1.63 days (SD: 1.31). Almost all (97%) the patients were treated with lopinavir-ritonavir with no recorded death. The median duration of hospital stay was 12 days (IQR: 9-13.5).

**Conclusion:**

In this preliminary analysis of the first COVID-19 cases in Nigeria, clinical presentation was mild to moderate with no mortality. Processes to improve promptness of admission and reduce hospital stay are required to enhance the response to COVID-19 in Nigeria.

## Introduction

Between November and December 2019, cases of a highly transmissible respiratory viral infection was observed in Wuhan, China and very soon was spreading to different parts of the world [1-3]. While the responsible virus was recognised as a coronavirus and initially referred to as the Wuhan Virus or 2019 novel Coronavirus (2019-nCov), further genetic studies revealed that it was a SARS-like virus and the International Committee on Taxonomy of Viruses has now named it SARS-CoV-2 and the disease complex caused by the virus as COVID-19 [4]. The action of the SARS-CoV-2 virus which results in COVID-19 disease is not fully understood [5]. The virus is a novel human infecting betacoronavirus likely originating from chrysanthemum bats [6]. The virus acts by using a densely glycosylated spike protein to enter host cells and then binds to the angiotensin-converting enzyme 2 (ACE2) receptor in humans. The ACE2 is typically expressed in type II alveolar cells in the lungs and may explain the clinical presentation of COVID-19 disease with upper and lower respiratory tract symptoms [6].

The epidemiological pattern of COVID-19 suggests an incubation period of five to fourteen days [6,7], with a recent case report suggesting as high as twenty-four days [8]. The mode of transmission of the SARS-CoV-2 virus is via respiratory droplets; but has also been found in blood and stool. Severe manifestations are more common in males, particularly in the elderly [2,6,9]. Clinical manifestations include fever, dry cough, fatigue, myalgia, headache, sore-throat, abdominal pain and diarrhea [2,5,6,9]. Further, laboratory and radiographic findings detail: elevated lactate dehydrogenase, prolonged prothrombin time, lymphopenia and chest CT scans demonstrating infiltrates with a ground glass appearance [2,6]. The treatment of COVID-19 remains largely supportive, with a number of cases requiring oxygen supplementation and intensive care [10-13]. Nonetheless, COVID-19 preventive measures will prove more effective and are less burdensome on the health system. In Nigeria, strict preventive measures are being taken to curb the spread of the ubiquitous virus, such as, isolation of infected and suspected cases, social distancing and health educational exercises on hygiene for the general population [14].

The Mainland Hospital in Yaba, Lagos State is the first hospital designated for the management of COVID-19 cases in Lagos and was the hospital that received and treated Nigeria´s first cases of the disease. As Nigeria rises to the challenge of curtailing COVID-19 [14], success will largely depend on a sound understanding of the epidemiologic and clinical profile of COVID-19 cases in our population and the case management approach. A locally focused analysis of treated cases in Nigeria might help identify the defining clinical characteristics and share experience with case management. This study therefore documents the presenting characteristics of the first 32 patients successfully treated at the Mainland Hospital, the treatment modalities and measures of outcome. Specifically, our objectives were to: describe the demographic characteristics and clinical profiles of the first 32 laboratory positive COVID-19 cases in Lagos State, Nigeria; describe the adopted main treatment parameters of these patients during their admission at the Mainland Hospital and document some parameters of outcome and identify areas of possible improvements in patient management.

## Methods

**Study site**: the cases reported in this study were patients treated at the Mainland Hospital, Yaba, Lagos, southwest Nigeria even though their places of residence were diverse in the Lagos Metropolis. The hospital is the first of five facilities in Lagos designated (as at time of the study) to isolate and treat COVID-19 patients being the foremost public infectious disease hospital in the state with funding support from the World Health Organisation (WHO) and the United States Agency for International Development (USAID). It has a 115 bedded admission facility with a female to male bed space ratio of 30:70, a dedicated biosafety laboratory, emergency operations centre and a biosecurity unit.

**Study design and population:** this was a retrospective study that used medical records of the first 32 patients admitted and discharged between February 27 and April 6, 2020. All patients were laboratory confirmed cases with naso-oropharyngeal and sputum specimens that tested positive for the SARS-CoV-2 virus by using real-time reverse-transcription-polymerase-chain-reaction (RT-PCR) assay for SARS-CoV-2 in accordance with the protocol established by WHO [15]. They were all discharged after two consecutively negative PCR-based SARS-CoV-2 virus tests.

**Data collection and analysis:** data was extracted from electronic medical records of the 32 cases as maintained by the case management team at the hospital. We obtained data on patients´ exposure history, clinical symptoms and signs, treatment modalities and outcomes. The outcomes of interest were death, promptness of admission process and duration of hospitalization. Severity of condition of patients upon presentation at the hospital was based on clinical symptoms and the need for oxygen and ventilation. A patient that was asymptomatic at presentation was classified as mild while a patient was classified as moderate if they presented with fever, cough, respiratory rate <30 breaths per minutes and peripheral capillary oxygen saturation (spO2) >90% for adults and >92% for children.

A patient with grunting respiration, respiratory rate >30 breaths per minute, spO2 <90% for adults and <92% for children requiring oxygenation was classified as severe while a patient with respiratory failure requiring mechanical ventilation was classified as critical. Our case definition was adapted from a handbook on clinical experience in China [16]. Data analysis was done using STATA version 15 (StataCorp, USA). Continuous variables were tested for the assumption of normality using the Shapiro Wilk test. Categorical variables were presented in frequencies and percentages, normally distributed continuous variables were presented as mean and standard deviation (SD) while non-normal continuous variables were presented as median and interquartile range (IQR).

**Ethics:** ethical approval was obtained from the Health Research and Ethics Committee of the Lagos State University Teaching Hospital. All patient data was anonymized and handled only by authorized personnel in order to ensure confidentiality.

## Results

The mean age of the patients was 38.1 years (SD: 15.5). Over half (53%) of them were aged 40 and above and most (66%) were male. Almost all patients (94%) had either recent travel history or contact with a confirmed case (Table 1). Table 2 presents the severity of patients´ condition and symptoms at presentation at the hospital. Three-quarters (75%) of the patients presented in moderately severe condition while 16% were asymptomatic. No patient presented in critical condition. Regarding the presenting symptoms of the patients, the most common ones were fever (59%) and dry cough (44%). Anosmia (loss of smell) and ageusia (loss of taste) was experienced by an equal proportion of patients (19% respectively). Over half (56%) of the patients were admitted within one to two days of having a positive result while 22% were admitted on the same day. The mean time between a positive test result and admission was 1.63 days (SD: 1.31). Upon admission, almost all (97%) the patients were treated with lopinavir-ritonavir. Only one patient (3%) was treated with chloroquine phosphate. No death was reported among the cases. Prior to discharge, the median interval between the first and second negative tests was 3 days (IQR: 2 - 3) with a range of 1 - 11 days. The hospital stay for the patients was between 6 to 24 days with a median duration of 12 days (IQR: 9 - 13.5) (Table 3).

**Table 1:** Age, sex and exposure history of patients (n=32)

Variables	Freq (%)
**Age group (years)**	
<1	1 (3.1)
1-19	3 (9.4)
20-39	11 (34.4)
≥40	17 (53.1)
Mean age, years (SD)	38.1 (15.5)
**Sex**	
Female	11 (34.4)
Male	21 (65.6)
**Exposure by travel or contact**	
Yes	30 (93.7)
No	2 (6.3)

**Table 2:** Severity of condition and presenting symptoms of patients (n=32)

Variables	Freq (%)
**Severity**	
Mild	5 (15.7)
Moderate	24 (75.0)
Severe	3 (9.4)
Symptoms*	
Fever	19 (59.4)
Dry cough	14 (43.8)
Shortness of breath	3 (9.4)
Sore throat	4 (12.5)
Runny nose	2 (6.3)
Sneezing	1 (3.1)
Body ache	5 (15.6)
Fatigue	7 (21.9)
Anosmia	6 (18.8)
Ageusia	6 (18.8)
Vomiting	2 (6.3)
Diarrhoea	2 (6.3)
Chest pain	2 (6.3)
Loss of appetite	2 (6.3)

* Multiple responses accepted

**Table 3:** Main treatment and outcome of patients (n=32)

Variables	Freq (%)
**Duration of Time Between Positive Testing and Admission**	
Same day	7 (21.9)
1-2 days	18 (56.3)
3-5 days	7 (21.9)
Meantime, days (SD)	1.63 (1.31)
Treatment*	
Lopinavir-ritonavir	31 (96.9)
Vitamin C	25 (78.1)
Paracetamol	16 (50.0)
Chloroquine	1 (3.1)
Median duration between 1st and 2nd negative tests, days (IQR)	3 (2-3)
Median duration of admission, days (IQR)	12 (9-13.5)

* Multiple responses accepted

## Discussion

This is the documentation of clinical presentation and management of the earliest confirmed cases of COVID-19 in Nigeria. The most common presenting symptoms were fever and cough, which is typical of the epidemiology of the disease and similar to findings in Asian [17,18] and European populations [19]. Although fever was the commonest symptom in our study, it did not occur in over a third of cases. This has implications in the identification of suspected cases by the use of patients´ body temperature. It has been suggested that undue emphasis should not be placed on temperature checks as a form of screening for COVID-19 [20].

A diminished ability to smell or taste, as reported by some patients in our study, has been recommended to be added to the list of primary symptoms for COVID-19 in USA and Britain as a result of the frequency of their occurrence [21]. A study conducted in the United States (USA) among COVID-19 positive and negative subjects revealed that these two symptoms showed the largest magnitudes of association with COVID-19 when compared to other symptoms [22]. Inclusion of loss of smell or taste in primary screening for COVID-19 could potentially be useful in the Nigerian context especially as these symptoms typically manifest in the early stage of disease or when the patient´s condition is mild [21,22]. However, further studies among a larger sample of subjects would be required to provide on-going evidence in the country.

The early confirmed cases of COVID-19 in Nigeria presented mostly in mild or moderately severe conditions. This is tandem with the known epidemiology of the disease. In China, where the pandemic began, 80% of the cases either did not have pneumonia or had a mild to moderate form [23]. One of the risk factors for severe disease is age 60 years and over and our population for study was a predominantly young one with only one case being over 60 years. None of the cases required the use of a ventilator, which is a scarce resource in Nigeria similar to other African countries. There are fewer than 2000 working ventilators in public hospitals across 41 African countries compared with more than 170,000 in USA [24]. There have been significant efforts to increase the number of ventilators in Nigeria in response to the pandemic from the government, donations and aid. However, challenges such as equitable distribution, skilled manpower, stable power supply and maintenance will need to be tackled as the pandemic progresses and cases with more severe conditions emerge.

Given that the epidemiology of COVID-19 in Nigeria has gradually transited from imported cases and their contacts to ongoing community transmission, it underscores the need to isolate and/or admit positive cases as soon as possible after testing. Less than a quarter of cases in our study were admitted on the same day a positive test was confirmed possibly because of logistic challenges at the onset of the outbreak. Modalities and processes improve as more experience is gained in tackling the disease.

The outcome for the early cases of COVID-19 was generally positive. There was no progression in severity of disease during admission and all 32 patients were discharged home recovered of the virus. The current evidence of benefit of using antiretrovirals to treat COVID-19 is of low certainty because of important limitations in previous studies [25]. Specifically, lopinavir-ritonavir, which was the treatment majorly used in our study, has shown to have no benefit over standard care in hospitalized patients with severe COVID-19 in one randomized clinical trial [26]. Thus, we recommend that the effectiveness of the use of the antiretroviral medication in our environment should be subjected to further robust and well-conducted research with a larger sample. There are not many studies yet that document the length of hospital stay for COVID-19. However, the median hospital stay in this study of 12 days (IQR: 9-13.5) was slightly higher than in Wuhan, China where the median hospital stay of 138 patients with COVID-19 pneumonia was 10 days (IQR: 7-14) [27]. Invariably, the length of time taken to conduct the laboratory test and get two negative results lengthened the hospital stay in our study; this should be weighed against the burden that increased hospital stay could place on limited medical resources especially as more admissions are warranted in the course of the pandemic. This study is limited by the small sample of cases from a single center at the beginning of an evolving pandemic and the results should be interpreted with caution. Also, this study was conducted in metropolitan Lagos and there is the likelihood that outcomes may differ in other states in the country that do not have as many resources in terms of manpower, money and materials.

## Conclusion

In this preliminary analysis of the first reported 32 cases of COVID-19 in Nigeria, clinical presentation was mild to moderate and all patients were discharged home with no mortality or complications. Additional research is required among larger samples of cases in the ongoing pandemic. Processes to improve promptness of admission and reduce hospital stay are required to enhance the response to COVID-19 in Nigeria.

### What is known about this topic

- COVID-19 is a new and ongoing pandemic and only a few studies (mostly among Asian and European populations) have been published that document the clinical picture, treatment and outcomes of COVID-19 patients.

### What this study adds

- Literature on COVID-19 is currently unfolding worldwide and study would contribute to the growing body of knowledge;
- In particular, this study helps in identify the defining clinical characteristics of COVID-19 and shares experience with case management in an African context.

## Competing interests

The authors declare no competing interests.
